# Abstract of Proceedings of the Buffalo Medical Association

**Published:** 1868-11

**Authors:** Thos. M. Johnson

**Affiliations:** Sec’y.


ART. II—*Abstract of Proceedings of the Buffalo Medical Association.*

TUESDAY EVENING, October 6th, 1868.

The meeting was called to order by the President. Members present—Drs. J. R. Lothrop, Trowbridge, Schuyler, Cronyn, Gay, Little, Samo, Rochester and Johnson.

DR. ROCHESTER related the following case: About six weeks ago a gentleman brought his son, aged twenty-one years, to me for advice and treatment. The patient had the appearance of being not more than fifteen or sixteen years old, was pale and feeble. He complained of severe pain on defecation. On examination found quite an extensive fissure of the anus, to which application of nitric acid was made. About four weeks after this the father came to request me to see the patient at his house, and said that the patient had not had an evacuation of the bowels within the last four weeks. This statement was undoubtedly correct. On examination found the intestines so distended with fecal matter and flatus that their course could be easily traced through the abdominal walls by palpation. With the assistance of Dr. Congar, gave chloroform, and on making examination of the rectum, found an obstruction about two and a half inches within the anus, which seemed like a fungoid growth. Undertook to pass a catheter, but failed to pass it above the obstruction. Then used a dilator and succeeded in making sufficient dilation to permit the passage of considerable flatus, which gave some temporary relief. The fissure had healed and was entirely well.

I regret that the result of the case cannot be given. On my giving an unfavorable prognosis, the patient was placed under the treatment of a quack, since which I have not known anything of the case. I regard it as a case of malignant disease, and report it because of the rarity of malignant disease in such young persons.

Dr. Lothrop thought that the situation of the disease, as described, would rather incline to the belief that the disease was cancer rather than stricture. Stricture was much more likely to be near the outlet, while a disease of the rectum causing narrowing, situated so high up as to be just within reach, in most cases would prove to be malignant. He had lately had under his care in the hospital, two cases, in which this distinction was shown. In one the narrowing of the intestine was high, so that the point of the finger would just reach it and was cancerous. In the other the narrowing was low, just within the sphincter and was a stricture proper.

The youth of the patient in Dr. R.'s case, did not forbid the idea of malignant disease. Cancer in very young persons was not so very rare. He had seen cases of cancer in very young persons, and could call to mind a medullary tumor in the temporal region in a young girl of about sixteen years.

In this connection he would inquire of the members present, what was the longest period of immunity from return, after removal of a malignant growth, which have come under the personal observation of any of them? His own experience had been unfortunate in this respect. In all the cases in which he had removed such growths there had been a return in a short time.

Dr. Cronyn said he had a year ago seen a boy, eight years old, who had asteosar coma, for which he removed a portion of the superior maxillary bone.

Dr. C. also related the case of a lady, twenty-six years of age, whom he saw two months ago for the first time. She had a short time before observed a small tumor upon the breast. She took cold at this time, when the tumor began to swell and became at the same time painful and assumed a purple color. The axillary gland and also the breast became much enlarged. The tumor opened spontaneously and discharged considerable blood. She died within four months of the first appearance of the tumor. There was no appearance of cachexia in the case.

Dr. Rochester said errors in diagnosis are easily made in cases of young persons. He sent a child of —— years to the Hospital of the Sisters of Charity last winter, of whom himself and other physicians thought she had malignant disease, but with iron, tonics, nourishing food, etc., she entirely recovered. Pressure was applied to some extent, but the recovery was due to the constitutional treatment and showed an error in diagnosis.

Dr. Rochester, in speaking of the prevalence of dysentery at the present time, would like to ask if the members present prescribed strychnia in this disease, and if so, with what results? He had given it to some extent, and usually in doses of five grains of the powdered nux vomica every six hours; continued it on one case four or five days. It had failed in every case in which he had employed it, and he had abandoned its use.

Dr. Gay said, we all administer strychnine in constipation, and believe it acts as a cathartic. Have never used it in dysentery, and should not expect good results from its use in that disease.

Dr. Rochester, I think Dr. Gay does not mean to say that strychnia is a laxative.

Dr. Gay, I state that it is a laxative, and I give it as such.

Dr. Cronyn said that he had given nux vomica in protracted cases of dysentery. Has used the solid extract in one-fourth or one-half grain doses, combined with opium or hen-bane, with good results. Has given one-fourth grain of nux vomica, one-half grain of opium, and one grain of quinine with good results. I suppose that but very few practitioners give strychnia alone as a laxative.

Dr. Lothrop had employed strychnia in diarrhœa with apparent benefit. He remembered one case in which the benefit from its use seemed quite marked. It was a case in which diarrhœa set in after an amputation of the thigh, accompanied with great prostration. The diarrhœa was so frequent and profuse that it seemed almost as if the beef essence taken into the stomach soon passed away by the rectum unchanged. Strychnia was given until it began to show its influence by muscular twichings, and at the same time improvement was apparent in the diarrhœa. He supposed that nux vomica, by its peculiar action upon the spinal cord, relieved constipation when it was caused by torpor or want of power, and would also benefit diarrhœa resulting from relaxation or deficient nervous control.

Dr. Gay would inquire if any of the members present were familiar with the use of *geranium maculatum?*

Dr. Lotrrop thought that *geranium maculatum* was a valuable astringent, but no better than kino, blackberry, or many others of the same class; in fact, no better than tannin, which is the basis of the astringent powers of all of them. In taste, however, it is not unpleasant, and on this account it might be preferred. He had used it considerably with children because its taste was less disagreeable than many other astringents. He thought tannate of bismuth much superior to it as an astringent. He had used the latter much more, and, as he thought, with benefit.

Dr. Gay said that he had used all kinds of astringents in diarrhœa and dysentery, and believed that the more astringents are used the worse it is for the patient, especially if given in large doses. He believed that in all chronic cases, if any astringent is to be given, geranium is the best.

Dr. Cronyn remarked that he believes that in a large majority of cases of dysentery that castor oil and twenty-four hours in bed is the best treatment.

Dr. Lothrop thought that if opium was needed, it was better to give it by the rectum. In this its general anodyne influence was equally good, while its local action in quieting tenesmus was more efficient. This was especially the case with children.

Dr. Gay spoke of a class of cases in which the patients seem to die from the sequelæ of dysentery. In these cases the patients seem to have been cured of the dysentery, we give them stimulus and expect them soon to be entirely well, but in a day or two or in a few days they die. What in these cases is the cause of death? The probable cause is some of the results of the dysenteric inflammation.

Dr. Cronyn said that a few days ago a medical friend of his took charge of a little patient which went on well for a few days, and recovery seemed almost certain, when quite suddenly reflex cerebro-spinal irritation came on and the patient died suddenly.

The Secretary read a communication from the Treasurer tendering his resignation. The resignation of the Treasurer was accepted and the Secretary directed to take charge of the books and papers.

Dysentery and diarrhœa were reported as prevailing.

Adjourned. THOS. M. JOHNSON, Sec'y.

